# Identification of Activating Mutations in the Transmembrane and Extracellular Domains of EGFR

**DOI:** 10.1021/acs.biochem.2c00384

**Published:** 2022-09-23

**Authors:** Anja Wagner, Edgar Galicia-Andrés, Magdalena Teufl, Lukas Gold, Christian Obinger, Peter Sykacek, Chris Oostenbrink, Michael W. Traxlmayr

**Affiliations:** † Department of Chemistry, Institute of Biochemistry, University of Natural Resources and Life Sciences, 1190 Vienna, Austria; ‡ Department of Biotechnology, Institute of Molecular Biotechnology, University of Natural Resources and Life Sciences, 1190 Vienna, Austria; § Department of Material Sciences and Process Engineering, Institute of Molecular Modeling and Simulation, University of Natural Resources and Life Sciences, 1190 Vienna, Austria; ∥ Department of Forest and Soil Sciences, Institute of Soil Research, University of Natural Resources and Life Sciences, 1190 Vienna, Austria; ⊥ Department of Biotechnology, Institute for Computational Biology, University of Natural Resources and Life Sciences, 1190 Vienna, Austria

## Abstract

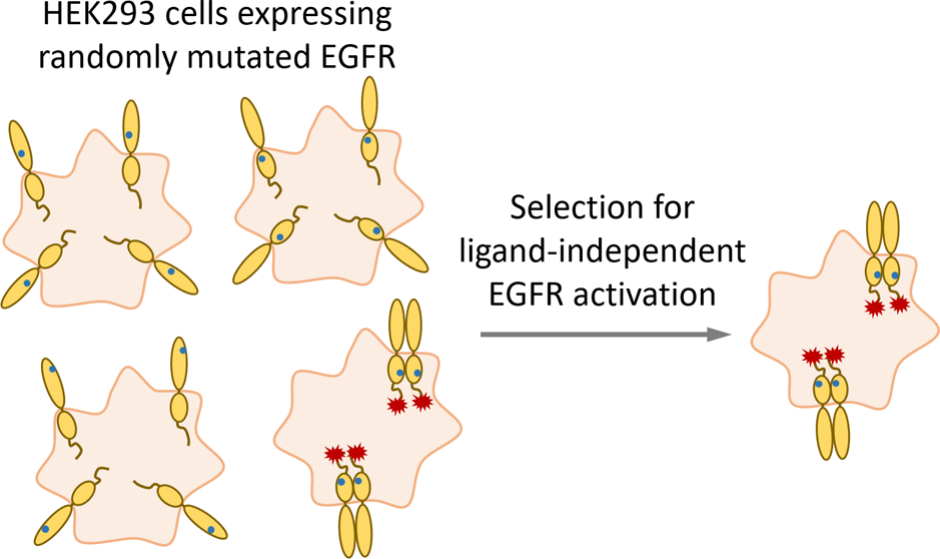

The epidermal growth factor receptor (EGFR) is frequently mutated in human cancer, most notably non-small-cell lung cancer and glioblastoma. While many frequently occurring EGFR mutations are known to confer constitutive EGFR activation, the situation is less clear for rarely detected variants. In fact, more than 1000 distinct EGFR mutations are listed in the Catalogue of Somatic Mutations in Cancer (COSMIC), but for most of them, the functional consequence is unknown. To identify additional, previously unknown activating mutations in EGFR, we screened a randomly mutated EGFR library for constitutive EGFR phosphorylation using a recently developed high-throughput approach termed PhosphoFlowSeq. Enrichment of the well-known activating mutations S768I, T790M, and L858R validated the experimental approach. Importantly, we also identified the activating mutations S442I and L658Q located in the extracellular and transmembrane domains of EGFR, respectively. To the best of our knowledge, neither S442I nor L658Q has been associated with an activating phenotype before. However, both have been detected in cancer samples. Interestingly, molecular dynamics (MD) simulations suggest that the L658Q mutation located in the hydrophobic transmembrane region forms intermolecular hydrogen bonds, thereby promoting EGFR dimerization and activation. Based on these findings, we screened the COSMIC database for additional hydrophilic mutations in the EGFR transmembrane region and indeed detected moderate constitutive activation of EGFR-G652R. Together, this study demonstrates that unbiased screening for activating mutations in EGFR not only yields well-established substitutions located in the kinase domain but also activating mutations in other regions of EGFR, including the extracellular and transmembrane domains.

## Introduction

The introduction of deep sequencing technologies has transformed our understanding of the development and genetics of cancer. More and more frequently, the entire cancer genome, its exome, or commonly mutated gene panels are sequenced to inform optimal treatment strategies.^1−3^ The epidermal growth factor receptor (EGFR) is a prominent example of a proto-oncogene that is frequently mutated in cancer, most notably non-small-cell lung cancer (NSCLC) and glioblastoma.^4−8^ Apart from being mutated, the *EGFR* gene is also frequently amplified in a range of human tumors.^8^ Due to the high incidence of EGFR dysregulation in human cancer, several EGFR-targeted therapies have been approved for clinical use. These EGFR-specific drugs can be largely divided into monoclonal antibodies (mAbs) targeting the extracellular domain (e.g., cetuximab, panitumumab, and necitumumab)^9^ and kinase inhibitors such as erlotinib, gefitinib, afatinib, and osimertinib, which block the enzymatic activity of the intracellular EGFR kinase domain.^10^ In many clinical centers, NSCLC samples are routinely screened for mutations in the *EGFR* gene^2,11^ since EGFR-mutant NSCLC has been shown to be more sensitive to EGFR-targeted kinase inhibitors.^12^

**Figure 1:**
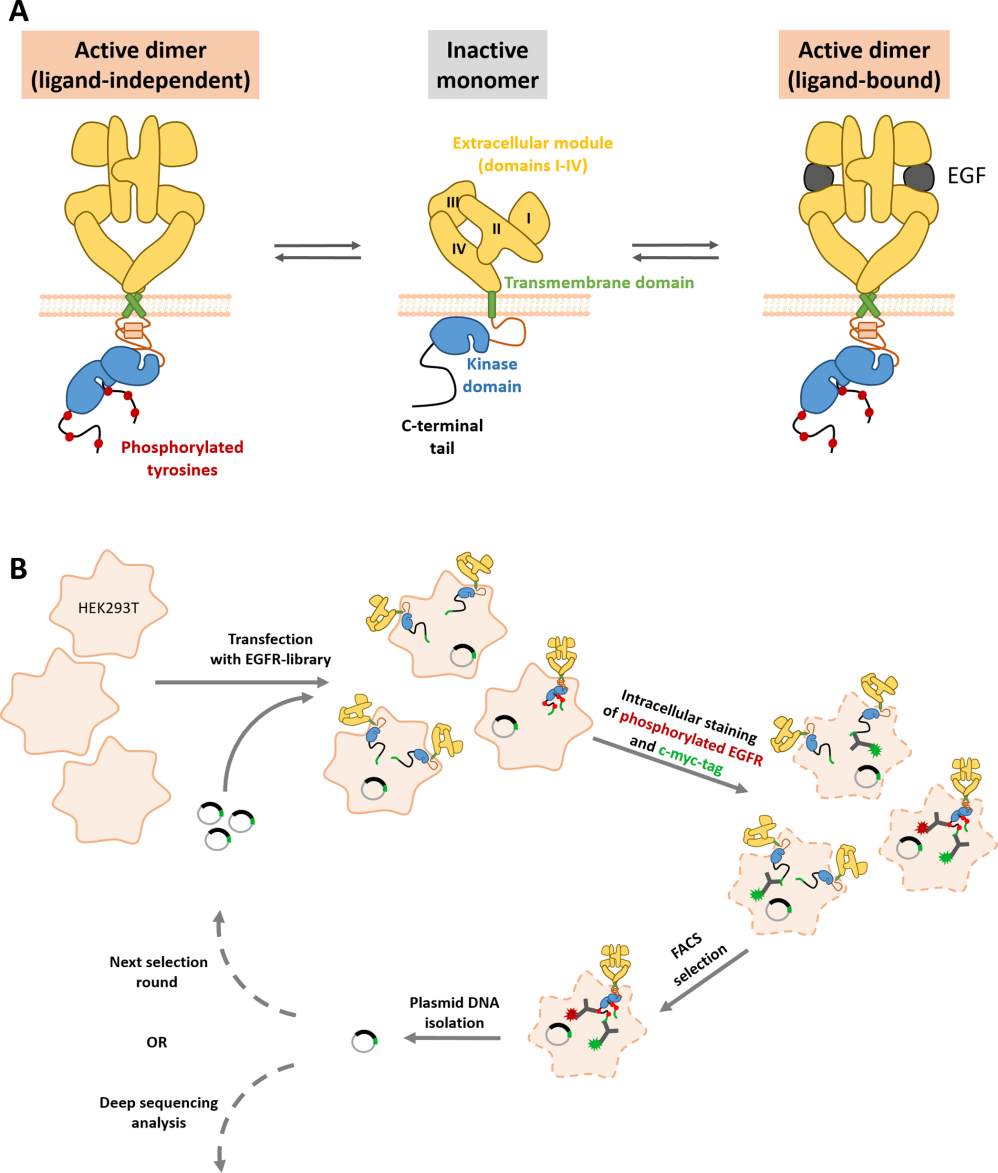
Schematic representation of the EGFR activation mechanism and of a PhosphoFlowSeq selection cycle. (A) In the absence of ligand, EGFR is predominantly present as a monomer with a tethered conformation in the extracellular module. Upon binding of EGF, the extracellular module switches to an extended conformation, thereby facilitating homodimerization and, as a consequence, activation of its kinase domains and autophosphorylation. Alternatively, EGFR can also become activated in a ligand-independent manner, particularly when being expressed at high densities. (B) The PhosphoFlowSeq selection cycle employed in the present study is based on transient transfection of HEK293T cells with a randomly mutated EGFR plasmid library. After 48 h, EGFR activation and expression are detected by intracellular staining, followed by flow cytometric sorting of cells expressing activated EGFR and plasmid DNA recovery from sorted cells. Some schematic components were adapted from ref (19).

EGFR is a member of the ErbB family of receptor tyrosine kinases (RTKs). It contains an extracellular ligand-binding module (comprising domains I to IV), a single transmembrane helix, as well as an intracellular module containing a juxtamembrane segment, a kinase domain, and a C-terminal tail (Figure 1A).^13^ Upon binding of a ligand such as EGF, the extracellular module of EGFR switches from a tethered to an extended conformation, which facilitates either homodimerization or heterodimerization with other ErbB family members (Figure 1A). Of note, in addition to the extracellular module, also the transmembrane helix, the juxtamembrane segment, and the kinase domain contribute to the dimerization interface (Figure 1A).^13−15^ Ultimately, this dimerization process allosterically transmits the signal from the extracellular growth factor to the intracellular kinase domains, which become activated and therefore phosphorylate specific Tyr residues on the C-terminal tail of EGFR.^8,15^ As an alternative to this canonical ligand-dependent activation mechanism, EGFR can also become activated in a ligand-independent manner, e.g., when being expressed at high densities^14^ or due to activating mutations found in NSCLC or glioblastoma (Figure 1A). Most of the activating *EGFR* mutations detected in NSCLC are located in the kinase domain, with L858R in exon 21 and small in-frame deletions in exon 19 being observed most frequently.^11,12^ In contrast, *EGFR* mutations found in glioblastoma are typically located in the extracellular module.^5^

Despite our detailed knowledge of the mutational landscapes found in cancer genomes, much less is known about the functional impact of many detected mutations. To address this limitation, numerous studies have been conducted with the goal to functionally characterize cancer-associated mutations. For example, Kancha et al. functionally analyzed 30 EGFR mutations repeatedly found in NSCLC samples, demonstrating that many, but not all of them conferred ligand-independent EGFR activation.^16^ Surprisingly, four of those mutations resulted in inactive EGFR, thus highlighting that the mere presence of a mutation—even if detected in a typical proto-oncogene like *EGFR*—does not provide sufficient evidence that the respective protein is activated. While those kinds of studies have significantly contributed to our understanding of the functional impact of mutations found in cancer samples, they can only cover a small subset of detected mutations. Thus, in contrast to the well-characterized, frequently occurring mutations, little—if any—phenotypic information is available for many rarely detected mutations, which are therefore often referred to as variants of unknown significance (VUS).^1^

More than 1000 distinct amino acid point mutations in the *EGFR* gene have been listed in the Catalogue of Somatic Mutations in Cancer (COSMIC).^17,18^ We hypothesized that, in addition to the well-known *EGFR* mutations, there would be additional activating mutations that have not been functionally tested yet due to their low frequency in human cancer. However, for obvious reasons, individual characterization of all *EGFR* mutations listed in COSMIC (more than 1000) is close to impossible, thus calling for high-throughput methods to identify activating mutations in *EGFR*. We have recently introduced a biochemically defined high-throughput assay termed PhosphoFlowSeq, which allows for direct analysis of kinase activities of randomly mutated *EGFR* libraries (Figure 1B).^19^ Briefly, HEK293T cells are transfected with an *EGFR* library generated by error-prone PCR. After applying selection pressure (e.g., by adding an EGFR-directed kinase inhibitor), the cells are stained intracellularly to detect both EGFR phosphorylation and EGFR expression, followed by flow cytometric enrichment of phosphoEGFR-positive cells. Since the intracellular staining step requires cell permeabilization, plasmids are isolated from sorted cells and *EGFR* genes are amplified by PCR. This enriched *EGFR* library can either be used for a second round of selection or be analyzed by deep sequencing (Figure 1B).

In our recent study, we demonstrated that PhosphoFlowSeq reproducibly enriches the resistance mutation T790M in several independent selection experiments performed in the presence of the EGFR-directed kinase inhibitor erlotinib.^19^ Since T790M is also by far the most frequently observed mutation in the *EGFR* gene upon erlotinib treatment of NSCLC patients,^20^ those experiments validated PhosphoFlowSeq as a reproducible method, enabling the identification of clinically relevant *EGFR* mutations. Of note, PhosphoFlowSeq harbors several critical advantages: (i) it directly screens for enzymatic activity (i.e., EGFR phosphorylation) instead of using a reporter gene or cell proliferation as a readout, thus making this approach less dependent on the intracellular signaling environment in the host cell used for the assay; (ii) due to an initial random mutagenesis step by error-prone PCR combined with the generation of large libraries, virtually the full mutational spectrum in the *EGFR* gene can be covered; and (iii) simultaneous detection of EGFR expression allows for compensation of expression-based biases on a single-cell level.

In the present study, randomly mutated EGFR libraries were screened for ligand-independent EGFR phosphorylation, i.e., for activating mutations. We observed enrichment of the well-known cancer-related mutations S768I, T790M, and L858R. In addition, mutations S442I and L658Q located in the extracellular and transmembrane domains, respectively, were also identified as activating mutations. Both S442I and L658Q have been detected in cancer samples, but, to the best of our knowledge, have not been shown to confer ligand-independent EGFR activation. Mechanistic studies at atomic scale using molecular dynamics (MD) simulations suggest hydrogen bonding within the hydrophobic environment of the plasma membrane as a potential molecular mechanism for L658Q-mediated dimerization and thus activation. This prompted us to screen the COSMIC database for additional hydrophilic mutations in the transmembrane domain of EGFR, identifying G652R as yet another mutation with an activating phenotype.

## Results

### Selection for Ligand-Independent EGFR Phosphorylation

Since the goal of the present study was the identification of activating mutations in *EGFR*, we slightly adapted the previously introduced PhosphoFlowSeq approach: Instead of creating selection pressure by adding a kinase inhibitor as had been done in the initial study,^19^ the EGFR library was screened for ligand-independent activation (Figure 1B).

**Figure 2:**
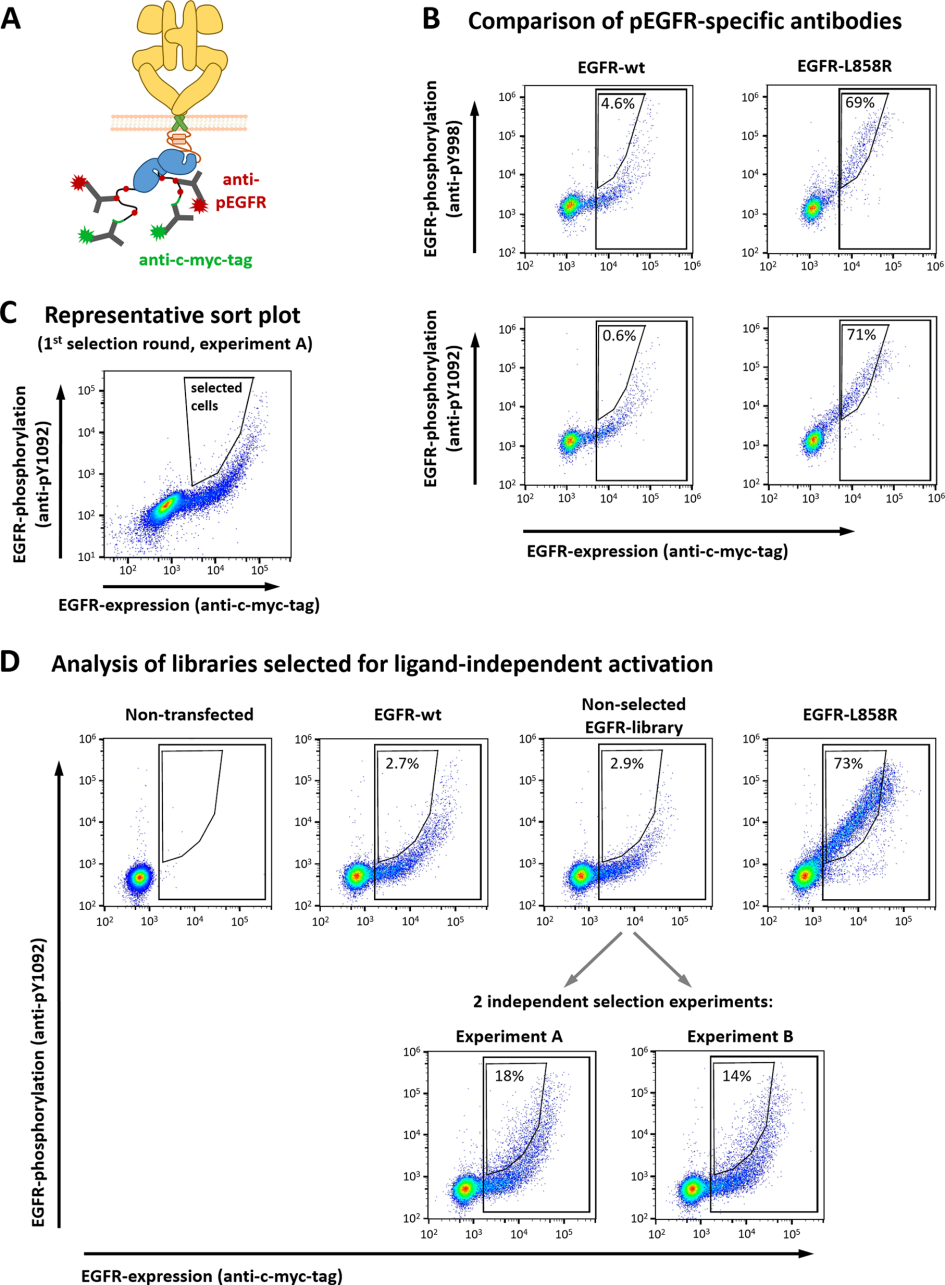
Selection of activating mutations in EGFR. (A) Schematic representation of the detection system used in the present study. EGFR expression was analyzed with a mAb recognizing a c-myc tag fused to the C-terminus of EGFR, whereas EGFR activation was detected with mAbs directed against phosphorylated tyrosines (pY) on the C-terminal tail of EGFR. (B) HEK293T cells expressing EGFR-wt or EGFR-L858R were analyzed for EGFR expression, as well as EGFR activation by detecting either pY998 or pY1092 on EGFR. The numbers in the plots indicate the percentage of cells in the small gates relative to those in the large rectangular gates. (C) Representative sort plot showing the first selection round of experiment A. (D) Two independent selection experiments were performed (experiments A and B, respectively), each of which contained two consecutive selection rounds. The data shown in this figure represent a comparison of the final, enriched library pools with the nonselected library, as well as various controls including EGFR-wt, EGFR-L858R, and nontransfected cells. The numbers indicate the percentage of cells in the small gate relative to the population in the large rectangular gate. Some schematic components were adapted from ref (19).

A key step in the PhosphoFlowSeq approach is the flow cytometric enrichment of phosphoEGFR-positive cells from a library expressing randomly mutated EGFR variants (Figure 1B). This step is based on the simultaneous intracellular detection of (i) phosphorylated Tyr residues (pY) on EGFR to assess EGFR activation and (ii) of a C-terminally expressed c-myc-tag to measure EGFR expression (Figure 2A). To optimize the selection efficiency, we first compared two mAbs recognizing different pY residues on EGFR (pY998 and pY1092, respectively). The pY1092-specific mAb consistently showed better separation of cells expressing the constitutively active mutant EGFR-L858R from those expressing wild-type EGFR (EGFR-wt) (Figure 2B). Thus, we anticipated that the selection of activating mutations from an EGFR library pool would be more efficient with the pY1092-specific mAb and therefore this mAb was chosen for the PhosphoFlowSeq selection experiments.

In agreement with the literature,^14^ we observed ligand-independent activation of EGFR-wt at high expression levels. That is, for EGFR-wt, the dependency of EGFR phosphorylation on EGFR expression was not linear, but exponential (Figure 2B). To minimize false positive enrichment of cells due to high expression levels, diagonal gates were used for selection (Figure 2C). As shown in Figure 2B, this gating strategy enables efficient separation of cells expressing EGFR with the activating mutation L858R from EGFR-wt-positive cells.

Using those optimized staining and gating strategies, two independent selection experiments were performed (termed experiments A and B, respectively), both of which included two rounds of selection. In both selection experiments, the theoretical diversity (10890 possible single-nucleotide mutations) was covered >20-fold (Table S3). Of note, enrichment of phosphoEGFR-positive cells was observed in both independent experiments when compared to the nonselected EGFR library (Figure 2D), strongly suggesting that activating *EGFR* mutations were successfully selected from the randomly mutated library.

### Sequence Analysis of Enriched Libraries

Next, both enriched library pools were analyzed by deep sequencing. In our previous study, we demonstrated that the required PCR amplification step of enriched *EGFR* genes resulted in some mutational noise. That is, even though a proofreading polymerase was used, certain mutations (especially C → T) were introduced at low frequencies during the amplification of enriched EGFR genes.^19^ Therefore, we have implemented three filtering steps in the sequence analysis, which were also applied in the present study: First, only mutations detected at a frequency of >1% after selection for ligand-independent activation were chosen. Second, mutations were only considered if they showed >8-fold higher frequency after selection for ligand-independent activation compared with a loss-of-function selection (no or low EGFR phosphorylation despite the presence of the ligand EGF). This filtering step greatly reduced the number of PCR artifacts, since those errors accumulated independently of the selection pressure (activating phenotype vs loss-of-function).^19^ Since the libraries selected in the present study (experiments A and B) were sequenced together with the previously published loss-of-function libraries^19^ in the same sequencing runs, those loss-of-function libraries were again used as reference datasets. Of note, the filter thresholds mentioned above were set such that previously known activating mutations, which were enriched in our screens, were not lost. So these thresholds are a compromise to achieve a reduction of mutational noise, while keeping known positive hits (which thereby served as “benchmarks”). Mutations remaining after these two filtering steps are presented in Table S1. As a third filter, only mutations listed in COSMIC at least once were considered. As a consequence, all mutations further analyzed in this study have been detected in a cancer sample at least once, suggesting that they may have clinical relevance.

**Figure 3:**
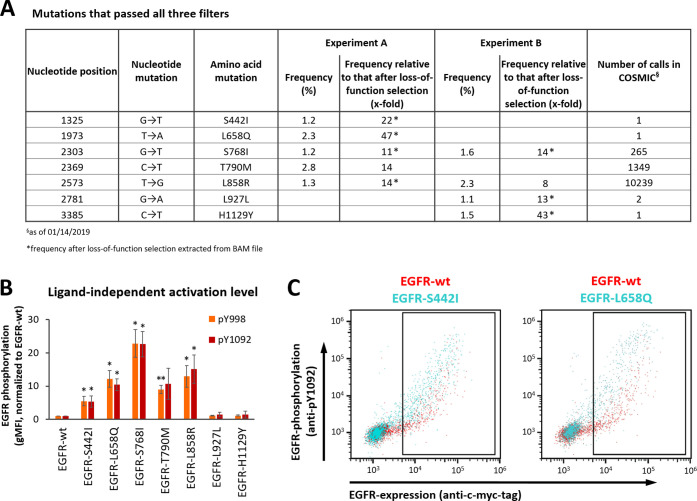
Analysis of EGFR mutations enriched in the two PhosphoFlowSeq selection experiments. (A) List of mutations that passed all three filters: (i) being detected at a frequency of >1% after selection for ligand-independent activation, (ii) showing >8-fold stronger enrichment in the selections for ligand-independent activation compared to loss-of-function selections, and (iii) being listed in the COSMIC database. (B) HEK293T cells were transiently transfected with plasmids encoding EGFR variants containing the mutations listed in (A). After 48 h, EGFR phosphorylation was analyzed using pY998- or pY1092-specific mAbs, as indicated. Only EGFR-expressing cells (being located in the rectangular gate shown in (C)) were included in the analysis. Average ± SD of geometric mean fluorescence intensity (gMFI) values of three independent experiments are shown. *p < 0.05, **p < 0.01, calculated using a two-tailed paired t-test. (C) Dot plot overlays of cells expressing EGFR-wt with those expressing either EGFR-S442I or EGFR-L658Q. Cells located in the rectangular gates were used for the analysis of EGFR phosphorylation levels depicted in (B). One representative of three independent experiments is shown.

After applying these three filters, a list of seven mutations remained (Figure 3A). The well-characterized variants *EGFR-L858R* and *EGFR-S768I* were enriched in both independent selection experiments (Figure 3A). Since both of them are known to confer ligand-independent activation,^6,7,16,21^ their reproducible enrichment further validated the PhosphoFlowSeq approach. We also detected *EGFR-T790M* in experiment A. While this mutation is primarily known for its ability to confer resistance to various EGFR-targeted kinase inhibitors,^8,20,22^ it has also been shown to trigger ligand-independent EGFR signaling.^6,16^

### Characterization of Enriched EGFR Mutations

To functionally characterize all seven enriched mutations, they were expressed in HEK293T cells and tested for their ability to trigger constitutive EGFR activation. In line with the literature mentioned above, EGFR-S768I, EGFR-T790M, and EGFR-L858R showed pronounced EGFR phosphorylation in the absence of ligand (Figures 3B and S1A). The mutations L927L and H1129Y did not elevate EGFR phosphorylation levels above EGFR-wt level, suggesting that they were accidentally carried over in the selection process and/or caused by polymerase errors during PCR amplification. However, we did observe constitutive activation for EGFR variants harboring the enriched mutations S442I or L658Q, which—to the best of our knowledge—have not been identified as activating mutations before (Figure 3B). Analysis using either pY998- or pY1092-specific mAbs yielded highly similar results, confirming that the outcome of these experiments was not critically dependent on the detected phosphorylation site on EGFR (Figure 3B).

Direct inspection of the dot plots demonstrates that the activating effects of both S442I and L658Q are observed across a broad range of expression levels (Figure 3C). That is, constitutive EGFR activation triggered by these mutations is not dependent on EGFR-overexpression. Moreover, quantification of surface expression levels revealed similar densities for all EGFR variants, except for EGFR-S442I, which was detected at lower levels (Figure S2A,B). These data further support the notion that the increased EGFR phosphorylation levels observed with the enriched EGFR-mutants are not primarily caused by elevated expression levels, but by an increased phosphorylation activity in the absence of ligand.

As expected, in the presence of the ligand EGF, the mutations identified in the PhosphoFlowSeq assay did not further increase EGFR phosphorylation levels when compared to EGFR-wt (Figure S1B). Taken together, seven mutations were identified after the two independent PhosphoFlowSeq selection experiments, of which five were confirmed to trigger ligand-independent EGFR activation (S442I, L658Q, S768I, T790M, and L858R).

### L658Q Potentially Promotes Transmembrane Dimerization

**Figure 4:**
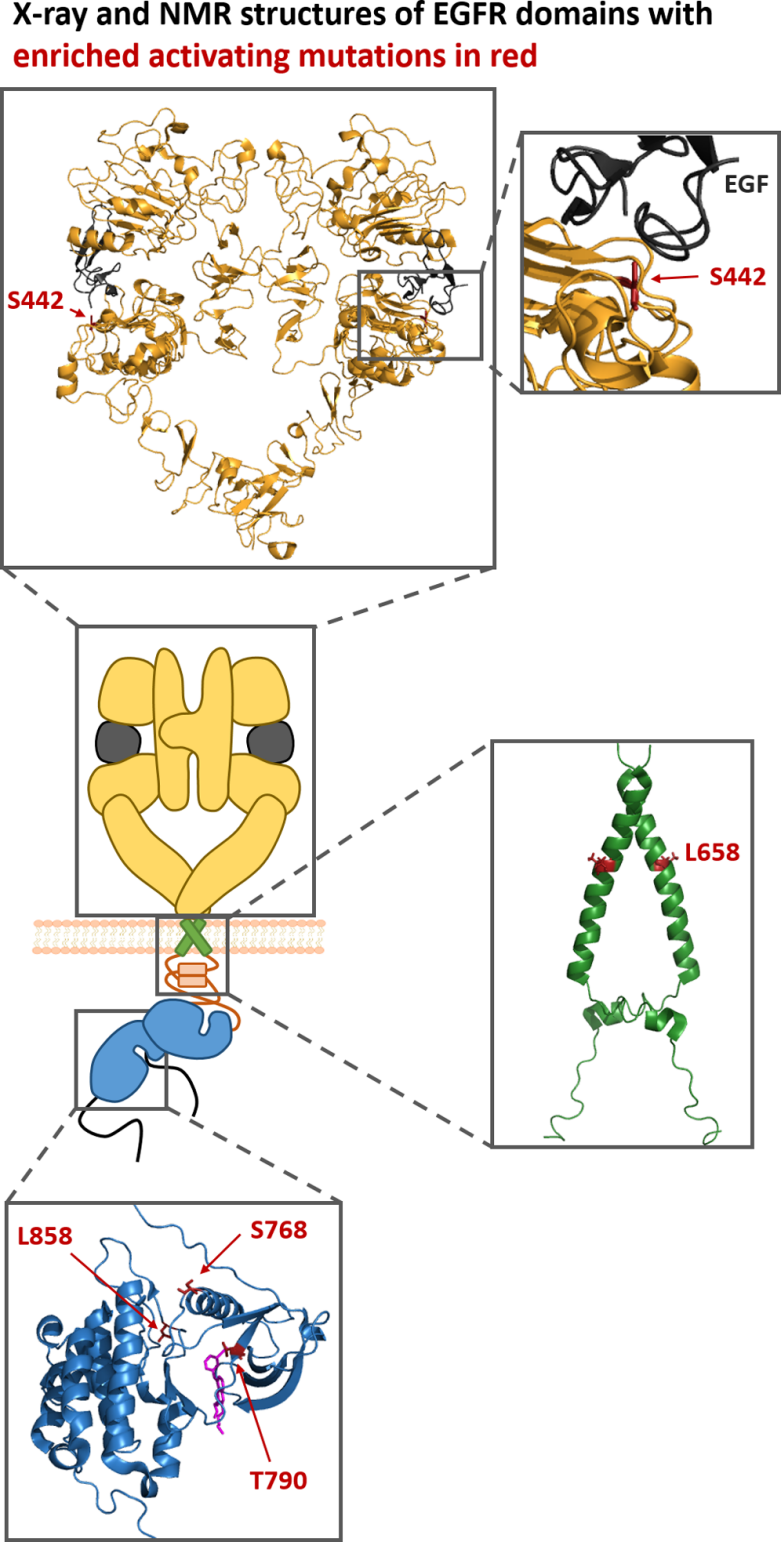
Location of enriched activating mutations within the EGFR structure. In the middle, a schematic structure of the ligand-bound EGFR dimer is shown. For various parts of this complex, crystal structures or NMR structures are depicted. Positions containing activating mutations enriched in this study are highlighted in red. Extracellular EGF-bound dimer: PDB-ID 3NJP;^24^ transmembrane domain dimer (including the intracellular juxtamembrane segment): PDB-ID 2M20;^14^ kinase domain bound to erlotinib (magenta): PDB-ID 1M17.^26^ The protein structures within this figure were generated using the PyMOL Molecular Graphics System. Some schematic components were adapted from ref (19).

Next, we inspected the location of those five identified activating mutations within the EGFR structure. While the well-characterized mutations S768I, T790M, and L858R are all positioned in the kinase domain, S442I and L658Q are located in the extracellular and transmembrane domains, respectively (Figure 4).

**Figure 5:**
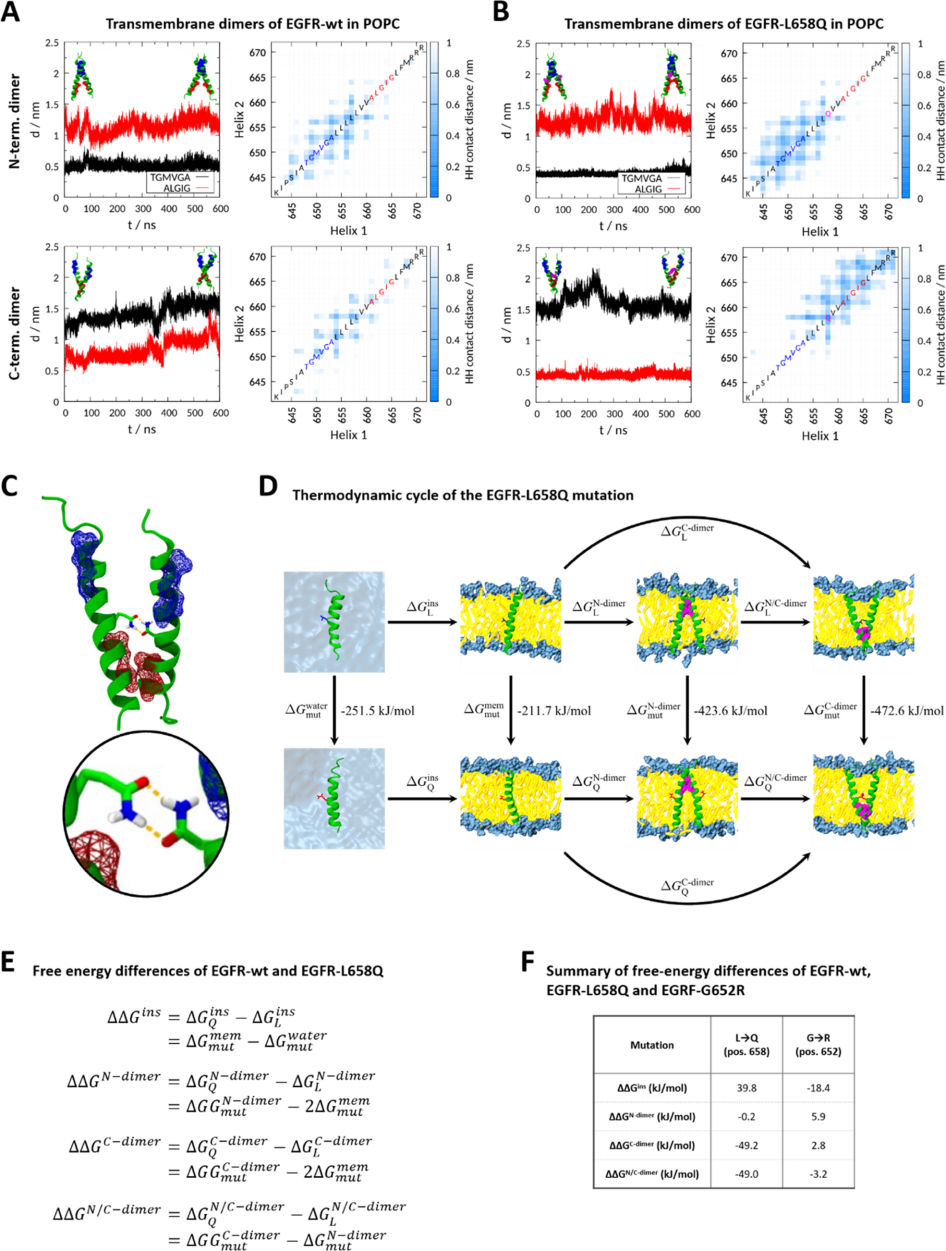
MD simulations of the transmembrane domains of EGFR-wt and EGFR-L658Q in a POPC bilayer. (A) Simulations of the N- and C-pose of EGFR-wt in POPC. (Left) Time series of the minimum distance between the GxxxG motifs of the two helices; snapshots of the initial and final conformations of the helices are shown with the motifs TGMVGA in blue and ALGIG in red. (Right) Average of the residue–residue contact distances between the two helices along the simulation. (B) Simulations of the N- and C-pose of EGFR-L658Q in POPC. (Left) Time series of the minimum distance between the GxxxG motifs of the two helices; snapshots of the initial and final conformations of the helices are shown with the motifs TGMVGA in blue, ALGIG in red and Gln residues in magenta. (Right) Average of the residue–residue contact distances between the two helices along the simulation. (C) Snapshot of the two helices of EGFR-L658Q hydrogen bonding between the Gln side chains. The motifs TGMVGA and ALGIG are shown in blue and red, respectively. (D) Thermodynamic cycle of peptides EGFR-wt and EGFR-L658Q in water and POPC bilayers. The Leu and Gln residues are represented as blue and red sticks, respectively, and the GxxxG motifs are shown in magenta. (E) Equations corresponding to the free-energy differences of the thermodynamic cycle for the L658Q mutation. These equations are equally applied for the G652R mutation. (F) Summary of free-energy differences of the thermodynamic cycle of L658Q and G652R. The calculated free energies correspond to the insertion process of the TM segment from water to a POPC bilayer membrane; the dimerization process of two helices on the GxxxG motifs close to the N-terminal (N-dimer) or the C-terminal end (C-dimer); and the pose change process from N- to C-pose. The alchemical mutation goes from Leu to Gln (at position 658) and Gly to Arg (at position 652). The protein structures within this figure were generated using VMD version 1.9.3.^27^

In particular, the emergence of the hydrophilic mutation L658Q close to the center of the hydrophobic transmembrane domain of EGFR caught our attention. To investigate the molecular mechanism of L658Q-mediated EGFR activation, we performed MD simulations on the EGFR transmembrane segment in lipid (POPC) bilayers using a previously published NMR structure (PDB-ID 2M20(14)). It has been suggested that dimerization of the EGFR transmembrane domain is mediated by GxxxG motifs, which are often found in transmembrane dimerization interfaces (it should be noted that in those motifs, G can be any amino acid with a small side chain).^14,23−25^ Compelling evidence suggests that upon ligand activation the EGFR transmembrane helix primarily dimerizes in its N-terminal region.^14,23,24^ This N-terminal part contains two overlapping GxxxG motifs, yielding the pattern “small-small-x-x-small-small” (TGMVGA). In our simulations with either EGFR-wt or EGFR-L658Q transmembrane segments, we also observed extensive contacts between the TGMVGA motifs and these N-terminal dimers were stable throughout the duration of the MD simulations (600 ns) (Figure 5A,B, for EGFR-wt and EGFR-L658Q respectively; plots on the top). However, it has also been proposed that—in particular in the absence of ligands—the EGFR transmembrane helix can also form C-terminal dimers, presumably via their C-terminal GxxxG motif (ALGIG).^14,23,28^ Interestingly, when we started our simulations with C-terminal dimers (Figure 5B, plots on the bottom), we observed extensive hydrogen bonding between the Gln residues of the L658Q variant (Figure 5C), suggesting that those intermolecular contacts favor dimerization. Additional simulations in DMPC bilayers confirmed the findings obtained in POPC bilayers, again showing hydrogen bond interactions between the Gln residues of the L658Q variant in C-terminal dimers (Figure S3B, plots at the bottom).

To test the hypothesis that dimerization of the transmembrane helices is enhanced by the L658Q mutation, we used the Crooks-Gaussian method^29^ and the Jarzynski equality^30^ to calculate and compare the Δ*G* of membrane insertion, dimerization, and pose change (*i.e.*, N- vs C-terminal dimer) of the wild-type and L658Q mutant according to the thermodynamic cycle in Figure 5D. The equations and the free-energy differences of each process are shown in Figure 5E,F, respectively. As expected, the hydrophilic nature of Gln disfavors the insertion of the transmembrane segment from water into a POPC membrane compared to Leu (ΔΔ*G*^ins^ = 39.8 kJ/mol; Figure 5F). However, while the formation of the N-terminal dimer is largely unaffected by the L658Q mutation (ΔΔ*G*^N-dimer^ = −0.2 kJ/mol), the mutation strongly favors C-terminal dimerization (ΔΔ*G*^C-dimer^ = −49.2 kJ/mol, Figure 5F), supporting the hypothesis that the L658Q mutation promotes dimerization of the EGFR transmembrane domain, thereby providing a potential mechanism for its activating phenotype observed in the cell assay.

### Identification of G652R as a Further Activating Mutation in the EGFR Transmembrane Helix

**Figure 6:**
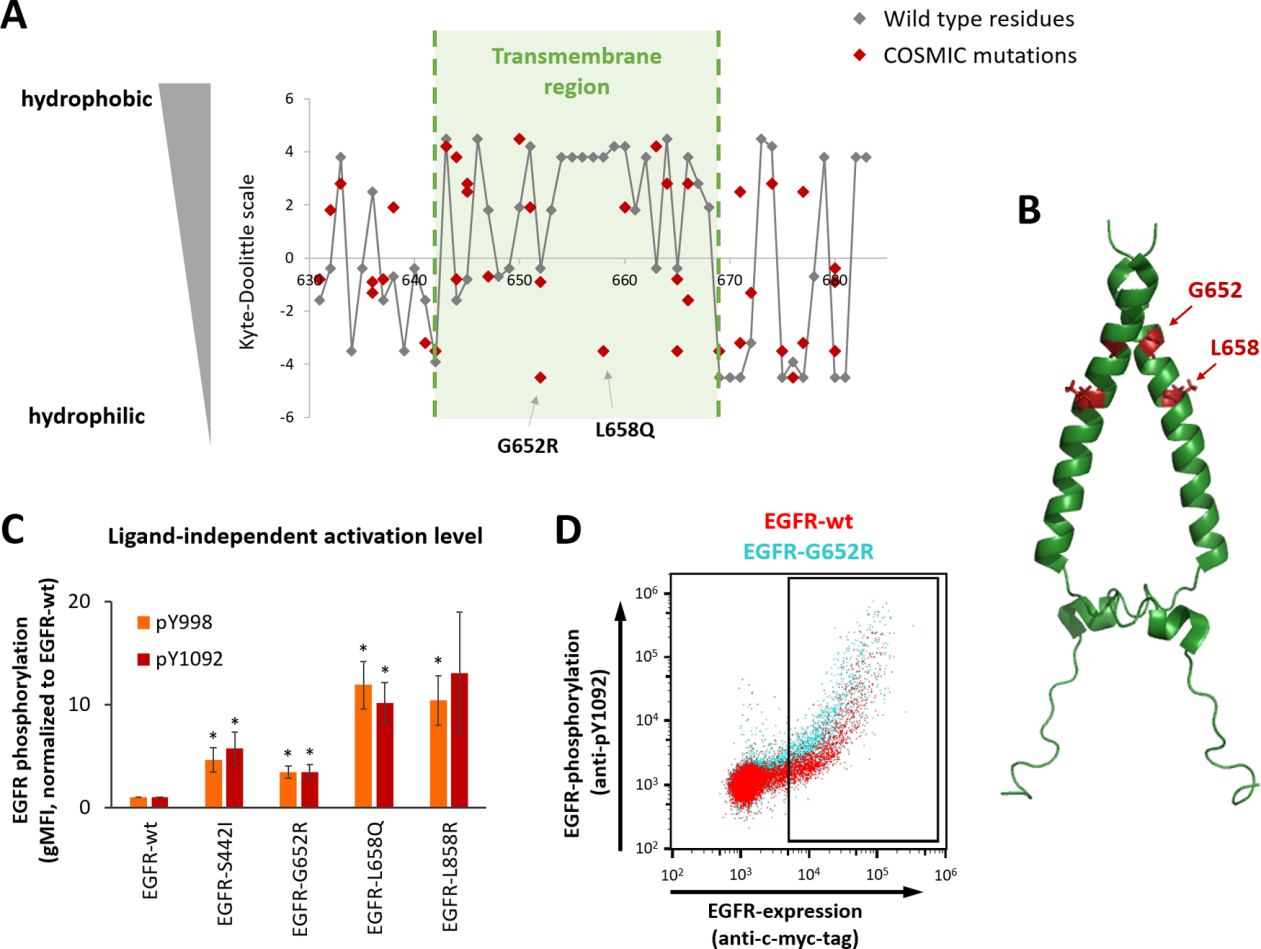
Characterization of the hydrophilic transmembrane mutation G652R. (A) All mutations located in the EGFR segment P631-L683, which were listed in the COSMIC database (as of Oct. 2020), are plotted on the Kyte-Doolittle hydrophobicity scale.^32^ Wild-type residues are indicated in gray, and mutations listed in COSMIC are shown as red dots. (B) NMR structure of the EGFR transmembrane domain and intracellular juxtamembrane segment (PDB-ID 2M20)^14^ with the positions G652 and L658 highlighted in red. The protein structure within this figure was generated using the PyMOL Molecular Graphics System. (C) HEK293T cells were transiently transfected with plasmids encoding various EGFR variants as indicated. After 48 h, EGFR activation was analyzed using pY998- or pY1092-specific mAbs, as indicated. Only EGFR-expressing cells (being located in the rectangular gate shown in (D)) were included in the analysis. Average ± SD of gMFI values of three independent experiments are shown. *p < 0.05, calculated using a two-tailed paired t-test. (D) Dot plot overlay of HEK293T cells expressing EGFR-wt or EGFR-G652R, respectively. Cells in the rectangular gate were analyzed with respect to their EGFR activation level to yield the values shown in (C). One representative of three independent experiments is shown.

Prompted by the results obtained with MD simulations, we screened the COSMIC database for the presence of further hydrophilic mutations within the transmembrane domain of EGFR. For that purpose, we plotted all COSMIC mutations located in the transmembrane helix (or in close proximity) on a hydrophobicity scale. As expected, only hydrophobic residues are present in the transmembrane domain of EGFR-wt (gray dots, Figure 6A). Similarly, most transmembrane mutations listed in COSMIC (red dots) are hydrophobic. However, we also observed a few exceptions, where hydrophilic mutations have been detected in the hydrophobic environment of the plasma membrane. Since residues located close to the membrane surface might snorkel out toward the hydrophilic head groups,^31^ we focused our attention to those located close to the center of the transmembrane domain (>5 residues from either end). This selection criterion ultimately yielded only two COSMIC-listed mutations: L658Q, which was described above as an activating mutation, as well as G652R (Figure 6A,B).

Indeed, when tested in the HEK293T-based system, G652R also conferred ligand-independent EGFR activation, albeit at lower levels compared with those triggered by L658Q or L858R. Again, highly comparable results were obtained with mAbs recognizing pY998 and pY1092, respectively (Figure 6C). Similar to S442I and L658Q, EGFR activation was not caused by elevated expression levels because (i) the activating effect was also observed at low or intermediate expression levels (Figure 6D) and (ii) surface expression levels were comparable between EGFR-wt and EGFR-G652R (Figure S2C). In the presence of the ligand EGF, phosphorylation levels of EGFR-G652R were comparable to or slightly lower compared with those of EGFR-wt (Figure S1C).

Next, we performed MD simulations for the G652R mutant as well, again showing the formation of N-terminal or C-terminal dimers (Figures S3C and S4). In contrast to L658Q, G652R favors the Arg residue over the Gly residue to be inserted into the membrane (ΔΔ*G*^ins^ = −18.4 kJ/mol; Figure 5F) because its hydrophilic, but relatively long side chain can snorkel out toward the hydrophilic head groups of the lipid bilayer. However, dimerization is not favored by the G652R mutation and, in fact, may even be slightly disfavored due to the mutual electrostatic repulsion of the positively charged side chains (ΔΔ*G*^N-dimer^ = 5.9 kJ/mol; ΔΔ*G*^C-dimer^ = 2.8 kJ/mol; Figure 5F). These data suggest that the activation mechanism of the G652R variant is different from that of L658Q and its elucidation will require further investigation in future studies.

Together, these data demonstrate that hydrophilic mutations located in the hydrophobic region of the EGFR transmembrane domain are rarely detected in cancer. However, the two that did show up in the COSMIC database both trigger ligand-independent EGFR phosphorylation.

## Discussion

In this study, we deployed PhosphoFlowSeq to screen a randomly mutated *EGFR* library for constitutive EGFR activation. Enrichment of the well-known activating mutations S768I, T790M, and L858R validated the screening strategy. In addition, we also selected the activating mutations S442I and L658Q, which, to the best of our knowledge, have not been functionally characterized before.

Importantly, both S442I and L658Q have been detected in cancer. According to COSMIC, L658Q was detected in a glioblastoma sample (COSMIC sample name: GB051T), whereas S442I was identified in a lung adenocarcinoma sample (COSMIC and ref (33)) and both were confirmed to be somatic mutations in those cases. Moreover, S442I was reported in a recent study, where it was found in a glioblastoma sample and listed as a mutation with “unknown” effect.^34^ Since most *EGFR* mutations detected in lung cancer are located in the kinase domain, in many previous studies, only exons 18–21 were sequenced.^2,35,36^ Thus, extracellular mutations such as S442I, as well as transmembrane mutations such as L658Q will be missed in such NSCLC studies. In this regard, in particular, the emergence of the activating mutation S442I in a lung adenocarcinoma sample deserves attention and calls for more comprehensive screening protocols covering the entire coding sequence of *EGFR*. Our unbiased approach yielded five activating mutations, of which two are located outside of the tyrosine kinase domain. Importantly, more comprehensive clinical studies also support this notion that *EGFR* mutations in lung cancer are not necessarily confined to the kinase domain. For example, Stein et al. analyzed 247 NSCLC samples and detected 43 *EGFR* mutations, of which 7 were located outside of the kinase domain.^11^ Thus, while the kinase domain indeed seems to be the mutation hot spot within the *EGFR* gene in lung cancer, mutations are also found in other parts, most notably in the extracellular domain.^11^ In contrast to NSCLC, it is known that in glioblastoma the majority of *EGFR* mutations are located in the extracellular domain.^5,37^

Interestingly, the mutation S442I is located in the epitopes of the clinically used antibodies cetuximab, panitumumab, and necitumumab.^38−40^ For cetuximab and panitumumab, S442 has even been shown to be engaged in hydrogen bonding with CDR-residues,^39,40^ raising the possibility that S442I not only acts as an activating mutation but that it might additionally impair binding of those EGFR-directed mAbs.

Another interesting observation was the identification of the hydrophilic mutation L658Q in the central region of the hydrophobic transmembrane helix. MD simulations suggest that the Gln side chains of this mutation form interdomain hydrogen bonds in the hydrophobic environment of the plasma membrane, thereby promoting C-terminal dimerization of the EGFR transmembrane segment. Several studies have shown that ligand-induced EGFR activation results in N-terminal transmembrane dimerization,^14,23,24^ which—at a first glance—might seem conflicting with our results (activation of EGFR-L658Q by C-terminal dimerization). However, it has also been demonstrated that ligand-induced EGFR activation was not impaired by a broad range of mutations in the N-terminal GxxxG dimerization motif, including full mutational scans of this region with Cys, Leu, and Phe mutations.^24^ This was confirmed by an independent study, where even simultaneous mutation of two key residues in the N-terminal GxxxG motif (G649I/A653I) did not affect EGFR activity significantly.^14^ Only simultaneous mutation of four residues within this motif reduced EGFR phosphorylation.^14^ Therefore, it was suggested that ligand-mediated EGFR activation results in N-terminal transmembrane dimerization, but this orientation may not be absolutely required for EGFR activation.^24^ This is in line with our results, which suggest that L658Q triggers EGFR activation through C-terminal transmembrane dimerization. It should be noted, though, that the ligand-independent phosphorylation level of EGFR-L658Q is comparable to those observed with other activating mutations (T790M or L858R; Figure 3B), but ∼4-fold lower compared with ligand-activated EGFR-wt (Figures 3B and S1B). Therefore, we hypothesize that N-terminal dimerization leads to more efficient EGFR activation than C-terminal dimerization, but EGFR showing C-terminal transmembrane dimerization is still much more active than the monomeric receptor.

Screening of the COSMIC database for additional hydrophilic mutations located in the transmembrane domain yielded G652R (Figure 6A,B), for which we also detected ligand-independent activation, but at a lower level. Together, these data suggest that the detection of a hydrophilic transmembrane mutation in EGFR in a cancer sample might be a first hint toward activated EGFR signaling, although this will certainly not be true for all hydrophilic transmembrane mutations. To the best of our knowledge, no activating EGFR transmembrane mutations have been associated with human cancer before. However, in rat neu, which is a homolog of human HER2 and closely related to EGFR, the oncogenic transmembrane mutation V664E has been described.^41^ The rat V664E mutation corresponds to the V659E mutation in human HER2, which has indeed been found in tumor samples of mainly lung cancer patients.^42−45^ Interestingly, MD simulations showed that the glutamic acid might be in fact protonated, forming an intermolecular hydrogen bond and thereby stabilizing the active dimer conformation.^46,47^ This would correlate with our findings regarding the L658Q mutation that also point toward increased stability between the two transmembrane monomers mediated by hydrogen bonding.

PhosphoFlowSeq was recently introduced and shown to reproducibly enrich the clinically most relevant drug resistance mutation T790M in response to erlotinib-resistance selections.^19^ The enrichment of well-known activating mutations in the present study (S768I, T790M, and L858R) further validates this screening approach. PhosphoFlowSeq harbors several critical advantages: First, screening for enzymatic activity (i.e., phosphorylation) instead of downstream signaling outcomes (e.g., proliferation) reduces the dependency on the intracellular signaling environment in the host cell. Second, an initial random mutagenesis step combined with flow cytometric high-throughput screening allows for comprehensive coverage of the mutational space in the target gene (Table S3). Third, simultaneous detection of EGFR phosphorylation and EGFR expression enables compensation of expression biases on a single-cell level. Especially with EGFR, which is known to become activated at high densities in a ligand-independent manner (Figure 2B, C and ref (14)), the simultaneous analysis of expression levels is a crucial advantage. In fact, given the strong activation of EGFR-wt at high expression levels (Figure 2B), it seems unlikely that selection solely based on EGFR phosphorylation would have yielded satisfying enrichments. This two-parameter detection strategy is analogous to the expression normalization that is routinely used in yeast surface display selections and known to considerably improve enrichment efficiencies.^48−50^

Of course, the present study also has some limitations. While several well-characterized (S768I, T790M, and L858R), as well as previously undescribed activating mutations (S442I and L658Q) were successfully enriched, some other reported activating mutations including A289V, G719S, and L861Q^4,5,16^ were not found after the filtering steps. Potential explanations include mutational biases in the original library (it should be noted, though, that the used error-prone PCR protocol has been shown to yield all types of nucleotide changes^51,52^), or a lower constitutive activation level of those other mutants, thus precluding efficient discrimination from EGFR-wt. Indeed, when we tested the known activating mutation EGFR-A289V^5^ in our assay system, we did observe slightly higher ligand-independent EGFR phosphorylation (compared with EGFR-wt), but at a lower level compared with the mutations that were identified in the present study (Figure S5). This suggests that A289V-mediated activation was detectable, but too weak for efficient enrichment during PhosphoFlowSeq selections. Another limitation of this study is the rare incidence of insertions and deletions after error-prone PCR, which was used for randomization of the *EGFR* gene. As a consequence, the well-known, short deletions in exon 19^12,16,36^ were not identified after PhosphoFlowSeq selections. Taken together, the mutations identified by PhosphoFlowSeq should not be regarded as the full set of activating mutations in EGFR. Nevertheless, among the four most frequently listed COSMIC mutations (L858R, T790M, L861Q, and S768I; as of July 2021) only L861Q was not identified in the PhosphoFlowSeq selections.

To sum up, we demonstrate that PhosphoFlowSeq facilitates the identification of activating mutations in *EGFR*. Apart from several well-known mutations, we identified two previously uncharacterized activating mutations in the transmembrane and extracellular domain of EGFR, respectively. We also provide a potential molecular mechanism of EGFR activation mediated by the hydrophilic transmembrane mutation L658Q and show that constitutive activation is also observed with another COSMIC-listed hydrophilic transmembrane mutation. Given the commercial availability of pY-specific mAbs for many other kinase substrates, we anticipate that PhosphoFlowSeq can be readily adapted to also study activating mutations and drug resistance mechanisms in other kinase genes.

## Methods

### Cell Culture

HEK293T cells were cultured in high-glucose Dulbecco’s modified Eagle’s medium (DMEM, Sigma-Aldrich) containing 10% fetal bovine serum (complete growth medium) and penicillin-streptomycin (both from Gibco, Thermo Fisher Scientific) at 37 °C and 5% CO_2_. The cells were routinely passaged every 3–4 days.

### Transfection and Application of Selection Pressure

The *EGFR*-containing plasmids (containing the initial error-prone PCR library, as well as *EGFR-wt* and *EGFR-L858R* as a control; EGFR-wt: UniProt-ID P00533) were generated in a previous study.^19^ The randomly mutated *EGFR* library was created by error-prone PCR using the GeneMorph II Random Mutagenesis Kit (Agilent Technologies) in our previous study.^19^ This error-prone PCR kit has been shown to yield all types of single-nucleotide changes;^51,52^ 24 h prior to transfection HEK293T cells were seeded in complete growth medium without antibiotics to reach 60–70% confluency at the point of transfection with *EGFR* plasmids. Transfection reactions were set up in Opti-MEM I reduced serum medium (Thermo Fisher Scientific) using the TransIT-X2 transfection reagent (Mirus BIO LLS) according to manual instructions. For the first selection round, EGFR plasmids were used at a concentration of 0.67 ng/mL, for the second round at 0.33 ng/mL, and for characterization of single mutants at 1 ng/mL. To improve transfection efficiency, an inert carrier pCTCON2-CD20 plasmid (generous gift from K. Dane Wittrup, MIT) was added at a concentration of 1 μg/mL.

To avoid activation of EGFR by growth factors in the medium, the complete growth medium was substituted by DMEM without serum 16 h before cell sorting (this step was done approximately 30 h after transfection).

### Antibody Staining

Cells were detached and resuspended with PBS. EGF in PBSA (PBS + 1% BSA; cold ethanol fraction, Sigma-Aldrich) was added to positive control samples at a final concentration of 100 ng/mL EGF. Untreated cells were substituted with equal amounts of PBSA and all samples were incubated for 5 min at 20 °C. The cells were fixated with 10 volumes methanol and incubated for 30 min at 4 °C. Samples were washed twice with PBSA, followed by transfer of a defined volume into a new tube to ensure that the concentrations of mAbs in the following staining steps are consistent between samples. Next, samples were stained with the primary rabbit mAb anti-pEGFR Tyr1092 (clone D7A5; Cell Signaling Technology; 1:800 final dilution) or rabbit mAb anti-pEGFR Tyr998 (C24A5; Cell Signaling Technology; 1:800 final dilution). Both primary antibodies were subsequently detected with 4 μg/mL polyclonal anti-rabbit IgG (H + L), F(ab′)2 fragment conjugated to Alexa Fluor 647 (Cell Signaling Technology). EGFR expression was detected intracellularly by adding 1.25 μg/mL anti-c-myc mAb (clone 9E10) conjugated to Alexa Fluor 488 (Thermo Fisher Scientific). Cell surface expression was analyzed with 2 μg/mL PE-conjugated anti-human EGFR antibody (clone AY13; BioLegend) using nonpermeabilized cells. All antibody incubation steps were done at room temperature for 30 min in the dark, followed by two wash steps with PBSA. EGFR surface expression was quantified using the BD Quantibrite PE Phycoerythrin Fluorescence Quantitation Kit (BD Biosciences) and cell viability was assessed by resuspending the cells in a 1:20 dilution of eBioscience 7-AAD Viability Staining Solution (Thermo Fisher Scientific) in PBSA after the antibody staining. All other samples were resuspended in PBSA only and kept constantly on ice until flow cytometric analysis or sorting.

### Flow Cytometry and Data Analysis

EGFR libraries were sorted either on a FACSAria Fusion cell sorter (BD Biosciences) or a MoFlow Astrios EQ cell sorter (Beckman Coulter). Comparisons of selected libraries and of single mutants were done on an LSR Fortessa, a FACSCanto (both BD Biosciences) or a CytoFlex S instrument (Beckman Coulter). FlowJo software (FlowJo, LLC) was used for the analysis of all flow cytometry experiments.

To analyze EGFR phosphorylation levels, EGFR-positive (i.e., c-myc-positive) cells were gated, followed by the analysis of the geometric mean fluorescence intensity (gMFI) of the phosphoEGFR-signal. After subtraction of background fluorescence (obtained from nontransfected cells in the absence of ligand), data were normalized to the level obtained with EGFR-wt without ligand, followed by calculation of average ± standard deviation (SD). Statistical analysis was performed using a two-tailed paired *t*-test (Microsoft Excel) and uncorrected *p*-values are reported.

### Preparation of Library Plasmids from Sorted Cells

Plasmid isolation from sorted cells was done using the QIAprep Spin Miniprep kit (QIAGEN) with the following modifications: To obtain a visible cell pellet, 2 × 10^5^ methanol fixed and further untreated HEK293T cells (nontransfected) were added to each pool of sorted cells. After centrifugation at 1500*g* and 4 °C for 5 min, the cells were resuspended in buffer “P2”, followed by the addition of 0.8 μg of pCTCON2-CD20 plasmid to improve plasmid recovery and 10 μL of Proteinase K (QIAGEN). After mixing, reactions were immediately incubated at 56 °C for 10 min and cooled down for another 4 min at room temperature. Buffer “N3” was added, followed by incubation on ice for 5 min and centrifugation at 18,000*g* and 4 °C for 10 min. All further plasmid isolation steps were done according to the kit’s manual. Isolated plasmids were PCR-amplified with Phusion polymerase (NEB) and primers EGFR_epPCR_fwd: CGCTGCCAAGCTTCCGAGCTCTCGAATTCAAAGGAGGTACCCACC and EGFR_epPCR_rev: AGGAGACAACTTCTAGAGGTCCTCTTCGGAGATCAGCTTCTGCTCAGATCCTCCGCCTCC) in the following two-step approach: The first amplification was done with 32 PCR cycles and a primer concentration of 0.5 μM, the second with 14 cycles and 0.075 μM. PCR reactions were supplemented with 10% DMSO. PCR products were restriction digested with XbaI and KpnI HF (both NEB) and ligated into the pSF-CMV-SV40 vector (Oxford Genetics) using Electroligase (NEB). Final ligation products were electroporated into 10-beta electrocompetent *Escherichia coli* (NEB) and library plasmids isolated from the liquid culture (LB with 50 μg/mL kanamycin) on the next day (yielding the pooled library plasmids). In addition, dilution series were plated on LB agar plates containing 50 μg/mL kanamycin immediately after the transformation process to estimate the diversity of the transformed *E. coli* culture from the number of counted colonies. Isolated plasmids were then either used for another round of selection or further prepared for Illumina sequencing.

### Illumina Sequencing

For Illumina sequencing 5 ng of library plasmids were used for amplification of the *EGFR* genes with an additional 14 cycle PCR as described above. PCR products were gel purified and sequenced by the Vienna Biocenter Next Generation Sequencing Core Facility (www.vbcf.ac.at) with either 50 bp single read or 125 bp paired-end on a HiSeq 2500 instrument (Illumina). Bioinformatic analysis was performed as described previously.^19^ In each library, the final average coverage within the *EGFR* coding region was above 100,000. Furthermore, the coverage was above 40,000 at each called nucleotide position.

### Site-Directed Mutagenesis

*EGFR* mutations were introduced using the QuikChange Lightning Site-Directed Mutagenesis kit (Agilent Technologies) according to manual instructions. The sequences of single clones were verified by Sanger sequencing.

### Simulation Details

We modeled the transmembrane segment by taking residues 1 to 30 for each monomer from the NMR structure with protein databank code 2M20.^14^ As the NMR structure sequence of the EGFR transmembrane domain deviates from ours and from those in the COSMIC database and UniProt (P00533), the sequence was altered by matching the L650 to M650 and I668 to M668 (the sequence used in the present study, as well as the UniProt sequence P00533 and the COSMIC sequence all contain M650 and M668). The corrected EGFR-wt sequence was used as base for all mutations. For the membrane bilayer, DMPC (1,2-dimyristoyl-sn-glycero-3-phosphocholine) was used to mimic the membrane conditions under which the NMR experimental structure was resolved, whereas POPC (1-palmitoyl-2-oleoyl-sn-glycero-3-phosphocholine) was used to mimic a mammalian plasma membrane, similar to Arkhipov et al.^23^ and Endres et al.^14^ The GROMOS 54a7 force field was used to describe the interaction of proteins and lipids and the SPC force field for water molecules. Simulations were performed using the Gromacs package version 2018.6. The temperature and pressure were kept constant at 310 K and 1 bar using the Berendsen thermostat and barostat with relaxation constants of 0.5 and 2.0 ps, respectively. A cutoff radius of 1.2 nm was used for the VdW interactions with energy and pressure corrections. The long-range electrostatic interactions were treated using the SPME algorithm with a cubic polynomial. The LINCS algorithm was used to constrain all bonds.

The initial conformation of the N-dimer corresponds to that from the 2M20 NMR structure, whereas for the C-dimer, it was determined by putting in contact both ALGIG motifs (the C-terminal dimerization motifs) at different helix–helix distances. At the optimal distance, the dimer’s minimum distance was smallest and the number of contacts was largest. In the case of mutations L658Q and G652R, residues were mutated using the Mutagenesis tool of PyMOL version 2.4.

## Notes

Illumina sequencing data generated during this study are available at the European Nucleotide Archive (ENA) (https://www.ebi.ac.uk/ena/browser/home; accession number: PRJEB49811). In addition, the tables with all called variants after NGS analysis are available in the Supporting Information.

## Supporting Information Available

Analysis of HEK293T cells expressing various EGFR variants; quantification of surface expression levels of EGFR variants; MD simulations in DMPC; simulations of the N- and C-pose of EGFR-G652R in POPC; analysis of EGFR-A289V; mutations remaining after the first two filtering steps; average values over the last 100 ns of MD simulations of helix–helix packing properties of Cα atoms and nonbonded interaction energies; and library diversities and coverage of the mutational space (PDF)
Mutations called in Experiment A (XLSX)
Mutations called in Experiment B (XLSX)

## Abbreviations

(EGFR): epidermal growth factor receptor
(NSCLC): non-small-cell lung cancer
(EGF): epidermal growth factor
(pY): phosphorylated tyrosine
(COSMIC): Catalogue of Somatic Mutations in Cancer
(MD): molecular dynamics
(DMPC): 1,2-dimyristoyl-sn-glycero-3-phosphocholine
(POPC): 1-palmitoyl-2-oleoyl-sn-glycero-3-phosphocholine
