# Supplementary material for: Identification of Activating Mutations in the Transmembrane and Extracellular Domains of EGFR
Source: Biochemistry. 2022 Sep 23;61(19):2049–62. doi: 10.1021/acs.biochem.2c00384 (PMC9535940; doi:10.1021/acs.biochem.2c00384)
Supplement: Supplementary file 1 — bi2c00384_si_001.pdf [file bi2c00384_si_001.pdf]

Supporting information

Identification of activating mutations in the transmembrane and extracellular domains of EGFR

Anja Wagner[1,2], Edgar Galicia-Andrés[3,4], Magdalena Teufl[1], Lukas Gold[1], Christian Obinger[1], Peter Sykacek[5], Chris Oostenbrink[3] & Michael W. Traxlmayr[1,*]

[1]Department of Chemistry, Institute of Biochemistry, University of Natural Resources and Life Sciences, Vienna, Austria; [2]Department of Biotechnology, Institute of Molecular Biotechnology, University of Natural Resources and Life Sciences, Vienna, Austria; [3]Department of Material Sciences and Process Engineering, Institute of Molecular Modeling and Simulation, University of Natural Resources and Life Sciences, Vienna, Austria; [4]Department of Forest- and Soil Sciences, Institute of Soil Research, University of Natural Resources and Life Sciences, Vienna, Austria; [5]Department of Biotechnology, Institute for Computational Biology, University of Natural Resources and Life Sciences, Vienna, Austria;

*to whom correspondence should be addressed: michael.traxlmayr@boku.ac.at

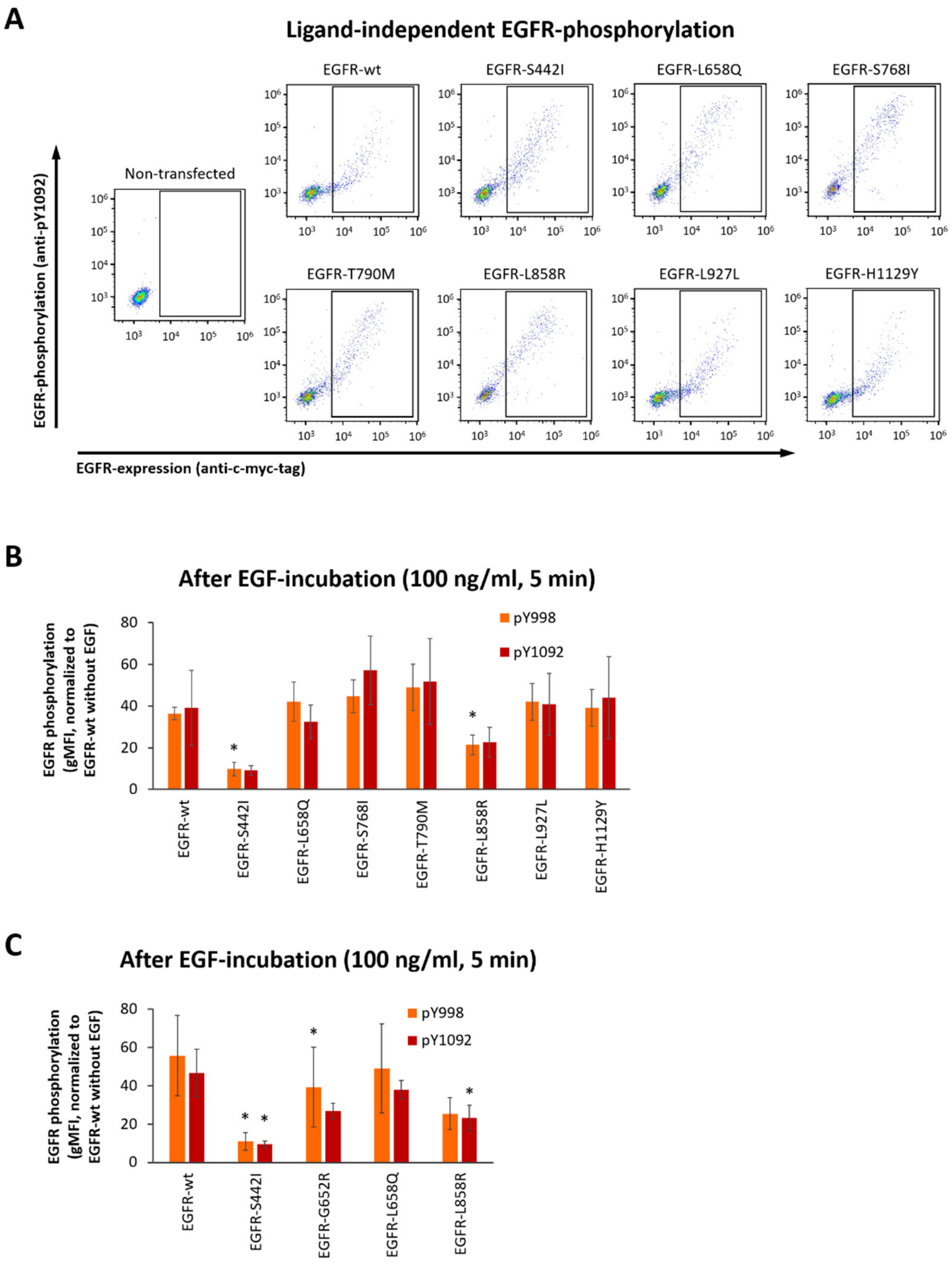

Figure S1

Analysis of HEK293T cells expressing various EGFR variants.

(A) HEK293T cells were transiently transfected with plasmids encoding enriched *EGFR* variants, as well as *EGFR-wt* as a control. After 30 hours, the supernatant was replaced with serum-free medium. After another 16 hours, cells were permeabilized with methanol and analyzed for EGFR phosphorylation and

EGFR expression using anti-pY1092 and anti-c-myc-tag, respectively. Cells in the rectangular gates were used to determine EGFR phosphorylation levels shown in Fig. 3B. The plots shown in this figure were also used to generate the dot plot overlays depicted in Fig. 3C. One representative of three independent experiments is shown.

(B) Same experiments as in Fig. 3B, but including a 5 min incubation with 100 ng/ml EGF immediately prior to methanol-fixation and intracellular staining. Averages ± SDs of gMFI values of three independent experiments are shown. *$p < 0.05$, calculated by using a two-tailed paired t-test.

(C) Same experiments as in Fig. 6C, but including an EGF-incubation step immediately prior to cell fixation and staining. *$p < 0.05$, calculated by using a two-tailed paired t-test.

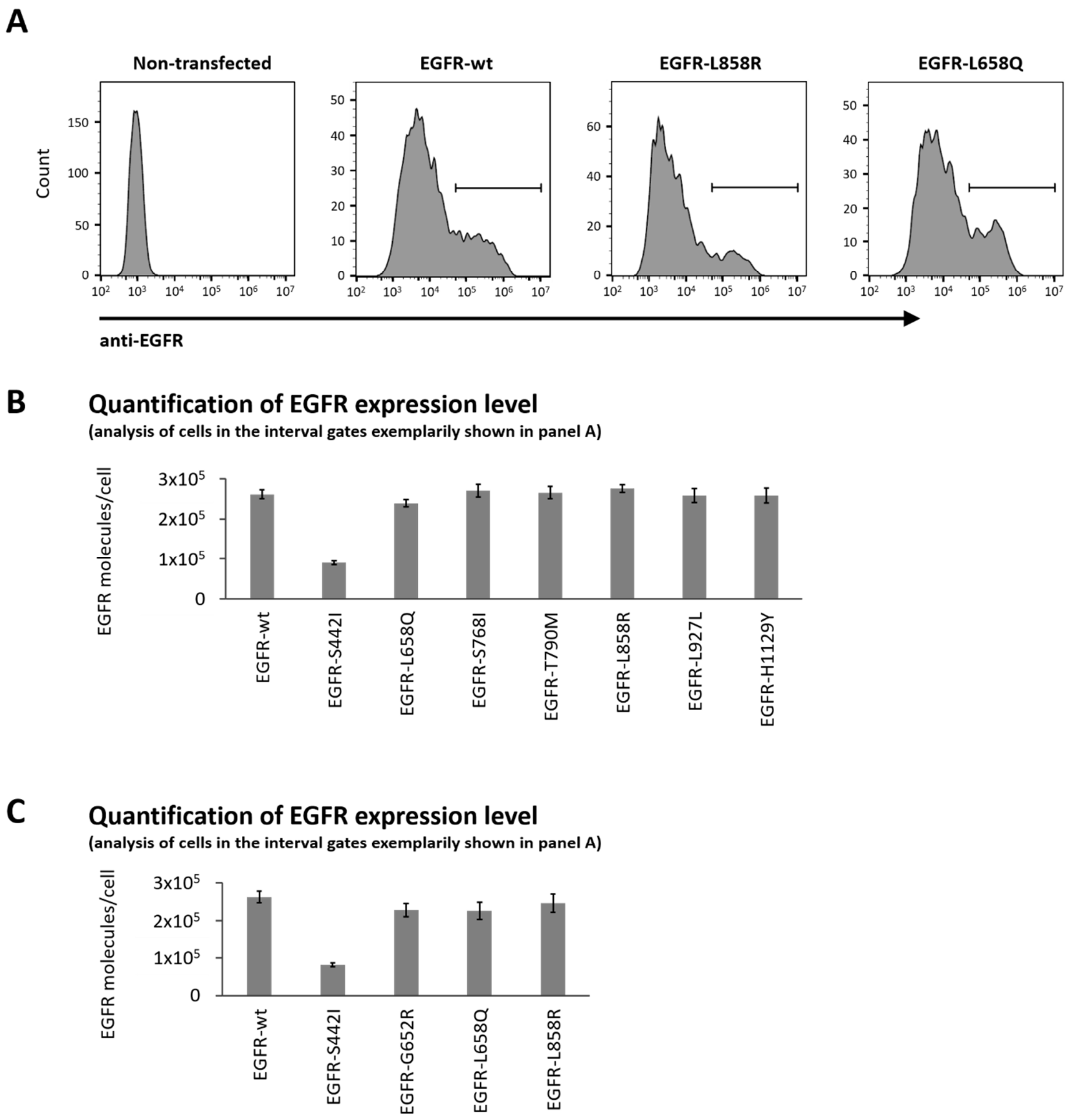

Figure S2

Quantification of surface expression levels of EGFR variants.

(A) HEK293T cells were transiently transfected with plasmids encoding EGFR variants. After 48 hours, EGFR surface levels were analyzed using anti-EGFR.

(B and C) EGFR surface expression levels were quantified by using Quantibrite^{TM} beads. Only EGFR-positive cells located within the interval gates in (A) were used for analysis. Averages ± SDs of three independent experiments are plotted. It should be noted that S442I is located in the extracellular domain and therefore it cannot be excluded that binding of the detection antibody is impaired, which might potentially explain the lower signal obtained for S442I.

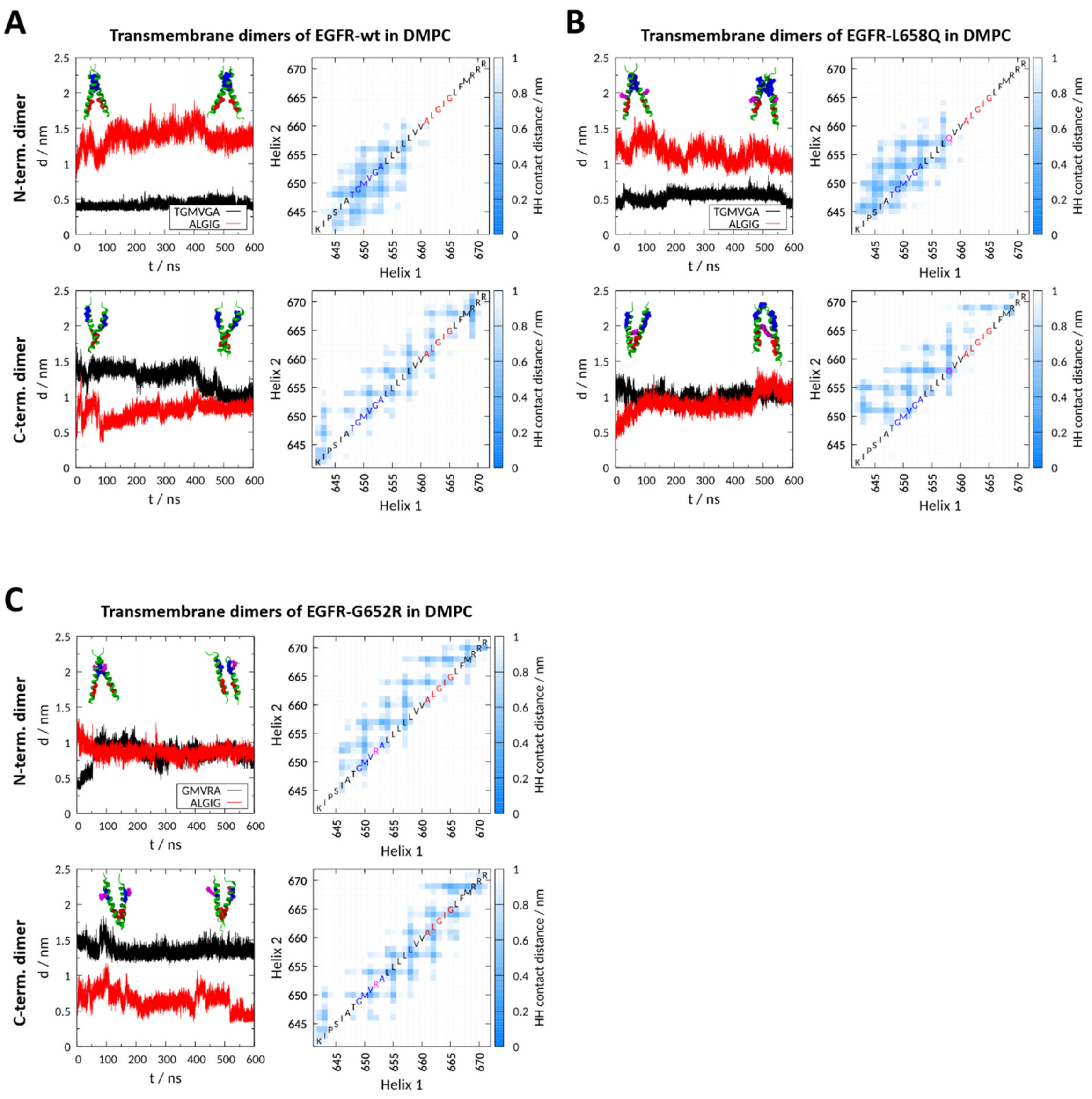

Figure S3

(A) Simulations of the N- and C-pose of EGFR-wt in DMPC. (Left panel) Time series of the minimum distance between the GxxxG motifs of the two helices; snapshots of the initial and final conformations of the helices are shown with the motifs TGMVGA in blue and ALGIG in red. (Right panel) Average of the residue-residue contact distances between the two helices along the simulation.

(B) Simulations of the N- and C-pose of EGFR-L658Q in DMPC. (Left panel) Time series of the minimum distance between the GxxxG motifs of the two helices; snapshots of the initial and final conformations of

the helices are shown with the motifs TGMVGA in blue, ALGIG in red and Gln residues in magenta. (Right panel) Average of the residue-residue contact distances between the two helices along the simulation.

(C) Simulations of the N- and C-pose of EGFR-G652R in DMPC. (Left panel) Time series of the minimum distance between the GxxxG motifs of the two helices; snapshots of the initial and final conformations of the helices are shown with the motifs GMVRA (this motif contains the G652R mutation) in blue, ALGIG in red and Arg residues in magenta. (Right panel) Average of the residue-residue contact distances between the two helices along the simulation.

The protein structures within this figure were generated using VMD version 1.9.3.[1]

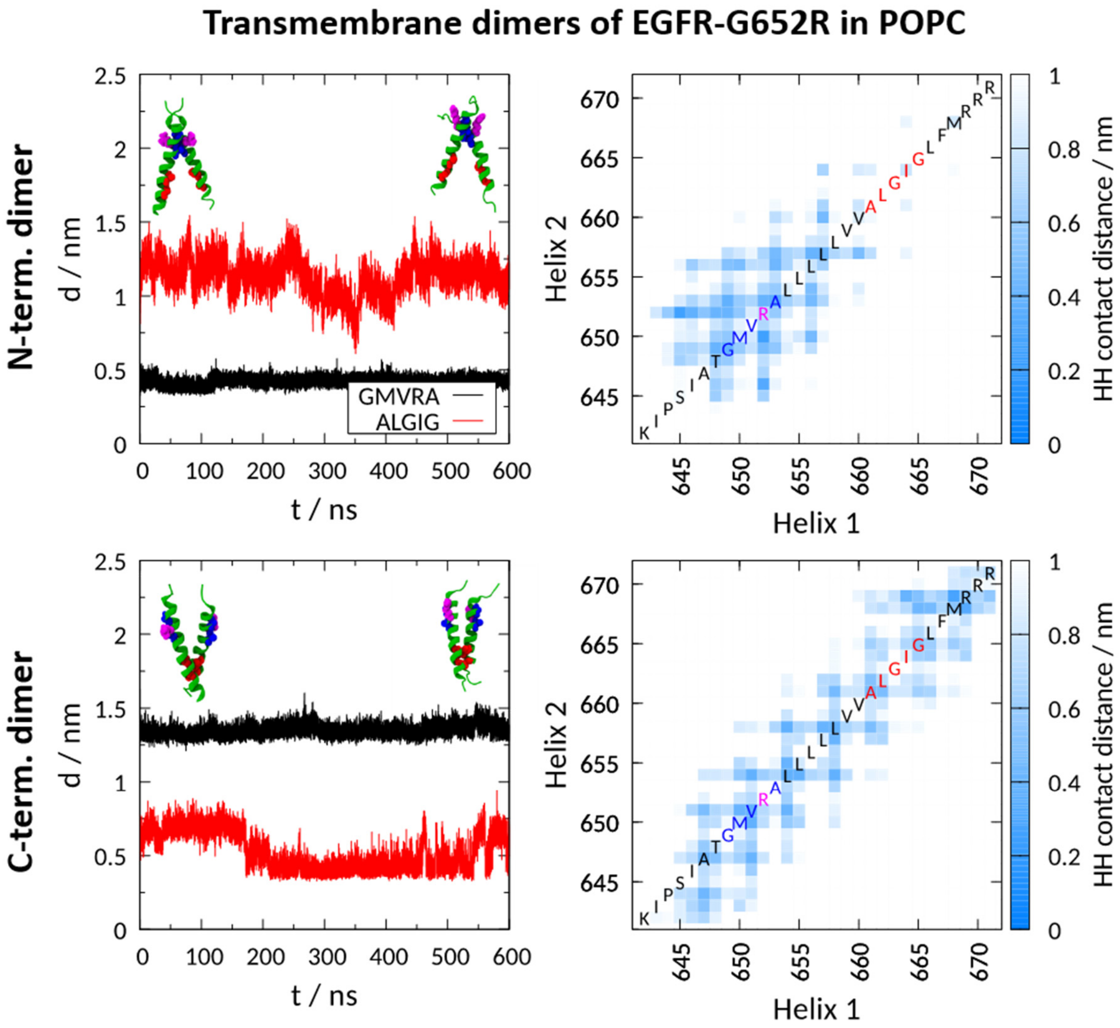

Figure S4

Simulations of the N- and C-pose of EGFR-G652R in POPC.

(Left panel) Time series of the minimum distance between the GxxxG motifs of the two helices; snapshots of the initial and final conformations of the helices are shown with the GxxxG motifs GMVRA (this motif contains the G652R mutation) in blue, ALGIG in red and Arg residues in magenta. (Right panel) Average of the residue-residue contact distances between the two helices along the simulation. The protein structures within this figure were generated using VMD version 1.9.3.[1]

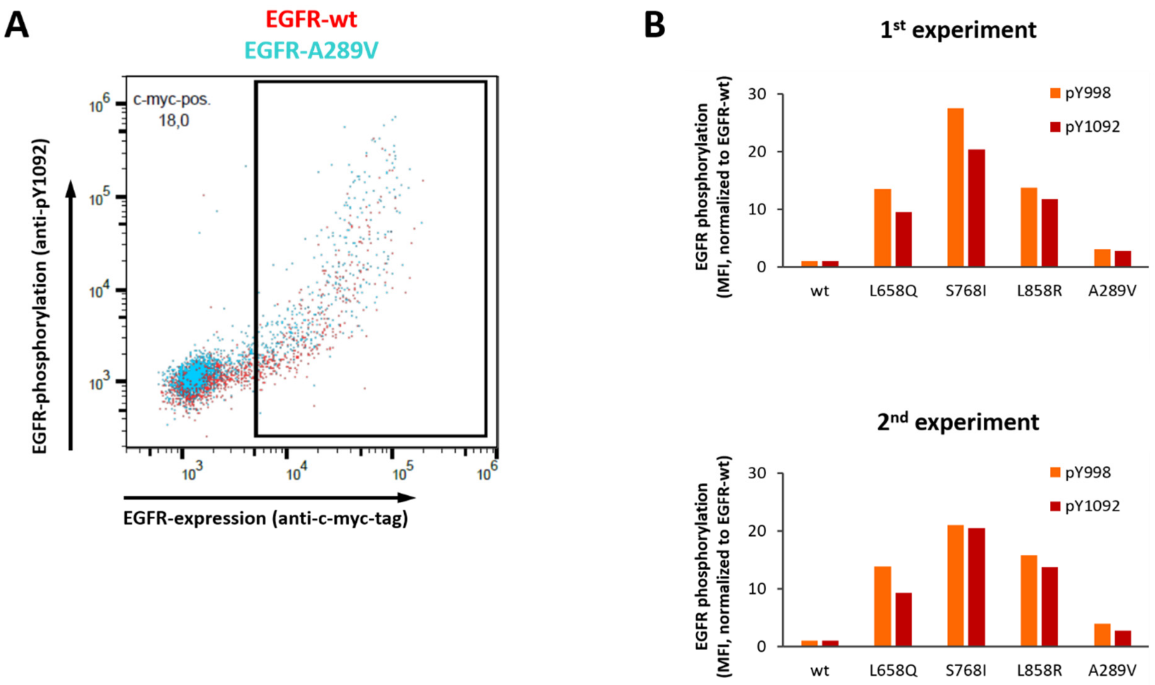

Figure S5

A289V confers weak, but detectable EGFR activation.

HEK293T cells were transiently transfected with plasmids encoding various EGFR-variants as indicated. After 48 hours, EGFR activation was analyzed using pY998- or pY1092-specific mAbs, as indicated.

(A) A dot plot overlay of HEK293T cells expressing EGFR-wt or EGFR-A289V, respectively, is depicted. Cells in the rectangular gate were analyzed with respect to their EGFR activation level to yield the values shown in (B). One representative of two independent experiments is shown.

(B) Cells located in the rectangular gate in (A) were analyzed with respect to their EGFR phosphorylation levels. Two independent experiments were performed and are presented individually.

Exp. A

Nucleotide position	Nucleotide mutation	Amino acid mutation	Frequency (%)	Frequency relative to that after loss-of-function selection (x-fold)	Number of calls in COSMIC§
1325	G→T	S442I	1.2	22*	1
1877	A→G	Y626C	1.0	16*	0
1973	T→A	L658Q	2.3	47*	1
2303	G→T	S768I	1.2	11*	265
2369	C→T	T790M	2.8	14	1349
2573	T→G	L858R	1.3	14*	10239
2676	C→T	T892T	1.3	9	0
2792	A→G	E931G	1.3	15*	0
3046	T→A	Y1016N	1.1	24*	0

§as of 01/14/2019

*frequency after loss-of-function selection extracted from BAM file

Exp. B

Nucleotide position	Nucleotide mutation	Amino acid mutation	Frequency (%)	Frequency relative to that after loss-of-function selection (x-fold)	Number of calls in COSMIC§
2	T→G	M1R	1.0	29*	0
143	T→A	F48Y	1.1	9*	0
321	C→A	I107I	1.0	15*	0
339	C→T	Y113Y	1.1	21*	0
473	A→G	N158S	1.3	15*	0
496	G→T	D166Y	1.1	11*	0
550	C→T	L184L	1.2	11*	0
587	A→T	N196I	1.5	45*	0
635	A→T	K212I	1.3	41*	0
753	C→A	C251→Stop	1.2	13*	0
781	A→T	K261→Stop	1.8	91*	0
909	T→A	D303E	1.1	17*	0
1058	C→T	A353V	1.4	22*	0
1305	G→C	Q435H	1.0	53*	0
1345	G→T	G449→Stop	1.6	10	0
1424	T→C	I475T	1.5	54*	0
1603	T→C	C535R	2.1	64*	0
1639	A→T	R547W	1.2	32*	0
1643	A→G	E548G	1.8	10*	0
2203	G→T	G735C	1.5	19*	0
2303	G→T	S768I	1.6	14*	265
2515	G→T	A839S	1.7	26*	0
2573	T→G	L858R	2.3	8	10239
2593	G→T	E865→Stop	1.3	22*	0
2781	G→A	L927L	1.1	13*	2
2849	G→A	C950Y	1.2	12*	0
3021	G→A	M1007I	1.6	38*	0
3258	C→A	F1086L	2.4	63*	0
3362	G→T	R1121I	1.1	9*	0
3385	C→T	H1129Y	1.5	43*	1
3451	T→A	F1151I	1.6	66*	0
3529	T→C	F1177L	1.2	27*	0
3597	G→T	R1199S	1.1	13*	0

§as of 01/11/2019

*frequency after loss-of-function selection extracted from BAM file

Table S1

List of mutations that were (i) detected at a frequency of >1% after selection for ligand-independent activation and (ii) which showed >8-fold stronger enrichment in the selections for ligand-independent activation compared to loss-of-function selections.

To calculate the ratio of the frequency after selection for ligand-independent activation vs. that observed after loss-of-function selection, only libraries analyzed within one sequencing run were compared with each other: The library obtained in experiment A of the present study was sequenced together (and therefore compared) with the loss-of-function library of experiment II of ref. [2], whereas the library obtained in experiment B of the present study was sequenced together (and compared) with the loss-of-function library of experiment IV of ref. [2]).

Structure	Membrane	Initial pose	R (nm)	Ω (deg)	τ_1 (deg)	τ_2 (deg)	ρ_1 (deg)	ρ_2 (deg)	Non-bonded energy (kJ/mol)
**2m20**	**DMPC**	N-dimer	1.1	-23.7	17.4	17.4	-1.6	-1.6	-
**TM-JM-WT**	**DMPC**	N-dimer	1.1	-25.5	13.0	13.8	24.1	17.7	-1019.6
**TM-WT**	**DMPC**	N-dimer	1.2	-33.7	23.1	22.1	52.8	-11.4	-95.4
		C-dimer	1.1	21.0	10.2	10.2	159.9	120.1	-181.3
	**POPC**	N-dimer	1.2	-38.8	21.1	21.2	19.5	21.5	-53.4
		C-dimer	1.2	35.0	17.7	17.4	86.0	125.9	-72.1
**TM-L658Q**	**DMPC**	N-dimer	1.1	-35.3	22.2	21.3	18.4	89.0	-116.4
		C-dimer	1.0	49.4	22.0	22.3	-146.4	-111.1	-159.3
	**POPC**	N-dimer	1.1	-35.2	23.0	22.8	17.2	14.4	-102.8
		C-dimer	1.2	25.3	18.0	17.8	169.5	108.5	-271.6
**TM-G652R**	**DMPC**	N-dimer	1.3	-12.7	7.3	7.0	59.0	19.1	-154.6
		C-dimer	1.0	24.8	12.8	13.1	164.9	79.8	-211.8
	**POPC**	N-dimer	0.9	-44.2	24.3	24.3	37.9	52.0	-167.6
		C-dimer	1.0	17.1	9.0	9.0	172.7	130.0	-246.0

Table S2

Average values over the last 100 ns of helix-helix packing properties of Cα atoms and non-bonded interaction energies.

To analyze the relative position and orientation between helices we used three points per helix defined by the Cα atoms corresponding to the center of mass (COM) of the helix, the COM of the top half of the helix and the COM of G649 and A653 Cα atoms related to the N-terminal motif, similar to Li et al.[3] The packing properties correspond to the interhelical distance between COMs of the helices, R, the interhelical crossing angle, Ω, the tilt angles of the i-helix with respect to the z-axis, τ_i and the relative rotation angle of the i-helix, ρ_i. The non-bonded interaction energy corresponds to the pairwise sum of the dispersive and electrostatic interactions of the helices.

Selection experiment	Selection round	Library diversity	number of screened EGFR-expressing cells	Coverage of theoretical diversity (10 890 single nucleotide mutations)	Coverage of library diversity	Cells sorted during selection	
						#	% of c-myc-positive cells
Experiment A	1st round	7.6E+05	3.0E+05	**28 x**	0.4 x	2.7E+03	0.9
	2nd round	2.7E+03	1.4E+05		**52 x**	4.7E+03	3.4
Experiment B	1st round	7.6E+05	5.7E+05	**53 x**	0.7 x	2.9E+03	0.5
	2nd round	2.9E+03	4.8E+04		**16 x**	1.1E+03	2.3

Table S3

Library diversities and coverage of the mutational space

Both selection experiments (A and B) comprised two selection rounds. The theoretical diversity (number of single nucleotide mutations) in the *EGFR* gene is 10890 (3 x 3630 bp). Sanger sequencing of individual clones randomly picked from the non-selected library revealed an average of ~1 nucleotide mutation per gene. The diversity of the original library was defined by the number of *E. coli* colonies obtained after ligating the randomly mutated *EGFR* genes into the vector and the following electroporation, yielding 7.6 x 10^5 individual transformants. However, in the first selection round the limiting factor was the number of screened, EGFR-expressing (i.e., c-myc-positive) cells. Thus, the coverage of the theoretical diversity was determined by dividing the number of cells screened in the first round by the theoretical diversity. In the second round the coverage was calculated by dividing the number of cells screened in the second round by the number of cells selected in the first round.

References

1. Humphrey, W., Dalke, A., and Schulten, K. (1996) VMD: visual molecular dynamics, *J Mol Graph 14*, 33-38, 27-38.
2. Wagner, A., Teufl, M., Gold, L., Lehner, M., Obinger, C., Sykacek, P., and Traxlmayr, M. W. (2021) PhosphoFlowSeq - A High-throughput Kinase Activity Assay for Screening Drug Resistance Mutations in EGFR, *J Mol Biol 433*, 167210.
3. Li, P. C., Miyashita, N., Im, W., Ishido, S., and Sugita, Y. (2014) Multidimensional umbrella sampling and replica-exchange molecular dynamics simulations for structure prediction of transmembrane helix dimers, *J Comput Chem 35*, 300-308.
